# Acute Osteochondral Fracture of the Metatarsal Head: A Report of Two Cases and Review Of Literature

**DOI:** 10.7759/cureus.30637

**Published:** 2022-10-24

**Authors:** Zied Missaoui, Mohamad K Moussa, Mohammad O Boushnak, Sarah Zahri, Ali Alayane

**Affiliations:** 1 Orthopedic Surgery, Grand Hôpital de l'Est Francilien - Site de Meaux, Meaux, FRA; 2 Orthopedic Surgery, Lebanese University, Faculty of Medical Sciences, Beirut, LBN; 3 Orthopaedics and Traumatology, Université libre de Bruxelles Hôpital Erasme, Brussels, BEL; 4 Orthopedics and Traumatology, Université libre de Bruxelles Hôpital Erasme, Brussels, BEL

**Keywords:** metatarsal fracture, metatarsophalangeal joint, open reduction internal fixation, osteochondral fracture, metatarsal head

## Abstract

Traumatic osteochondral fractures of metatarsal heads are rare injuries and are scarcely reported in the literature. Their classification and modalities of treatment remain unclear. Herein, we report two cases of traumatic fractures of the articular surfaces of the metatarsal heads in two young patients in which two different modalities of surgical treatment were used to achieve anatomic reduction and congruity of the metatarsophalangeal joints. The postoperative period was uneventful, and good functional and radiological outcomes were achieved in both patients. In this report, we review the literature for similar cases and discuss the available treatment options and their associated complications.

## Introduction

Traumatic metatarsal head osteochondral fractures (MHOCF) are uncommon injuries which are rarely described in the literature [1,2]. Reported MHOCFs are usually nontraumatic and secondary to stress fractures, Frieberg’s disease, or fatigue lesions [3,4]. They are usually associated with adjacent metatarsal bone fractures [5]. MHOCF classification and treatment modalities remain unclear. The most important prognostic factor in obtaining good long-term functional results is the restoration of the congruity of the metatarsophalangeal joint (MTPJ). This restoration decreases the risk of metatarsal head necrosis [1,6]. The aim of this paper is to report two additional acute traumatic slipped-retroverted MHOCFs treated with open reduction internal fixation by using two different surgical techniques.

## Case presentation

Case 1: Isolated MHOCF

**Figure 1:**
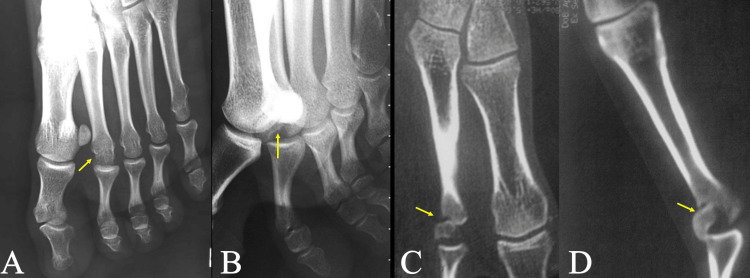
(A, B) Anteroposterior and lateral plain radiographs of the left foot showing the intraarticular slipped retroverted osteochondral fracture of the 2nd metatarsal head (yellow arrows). (C, D) Computerized tomography images showing displaced retroverted osteochondral fragment of the second metatarsophalangeal joint (yellow arrows).

A 19-year-old healthy male presented at the clinic with left foot pain and inability to bear weight due to left foot trauma. His medical history goes back to one week prior to the presentation, when he injured his left foot during a handball game. Physical examination showed left foot swelling, ecchymosis, and tenderness on palpation over the second MTPJ with intact overlying skin. A restricted range of motion of the left second metatarsophalangeal joint was also noticed. The neurovascular examination was normal. Radiographs of the left foot showed isolated closed dorsally displaced second MHOCF (Figures 1A-1B). A computerized tomography scan of the left foot confirmed the diagnosis (Figures 1C-1D).

**Figure 2:**
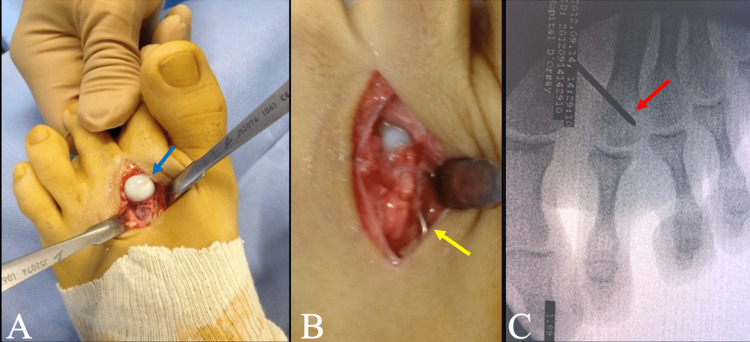
(A) Intra-operative photos showing rolled back osteochondral fragment (blue arrow), (B) anatomical reduction and internal fixation of the fracture with K-wires (yellow arrow), (C) Intraoperative fluoroscopy after fixation of the fragment by a K-wire (red arrow).

Under spinal anesthesia, a 3 cm longitudinal dorsal incision over the second web space was done. Retraction of the extensor tendons followed by dorsal second metatarsophalangeal joint capsulotomy showed the dorsally displaced retroverted osteochondral fragment (Figures 2A-2B). The cartilage was hard and intact with good bleeding subchondral bone confirming the diagnosis of traumatic second metatarsal head fracture. Under fluoroscopy guidance, the reduction of the articular fragment was done with gentle digital traction in order to protect the articular cartilage followed by 1.5 mm antegrade K-wire fixation (Figure 2C).

**Figure 3:**
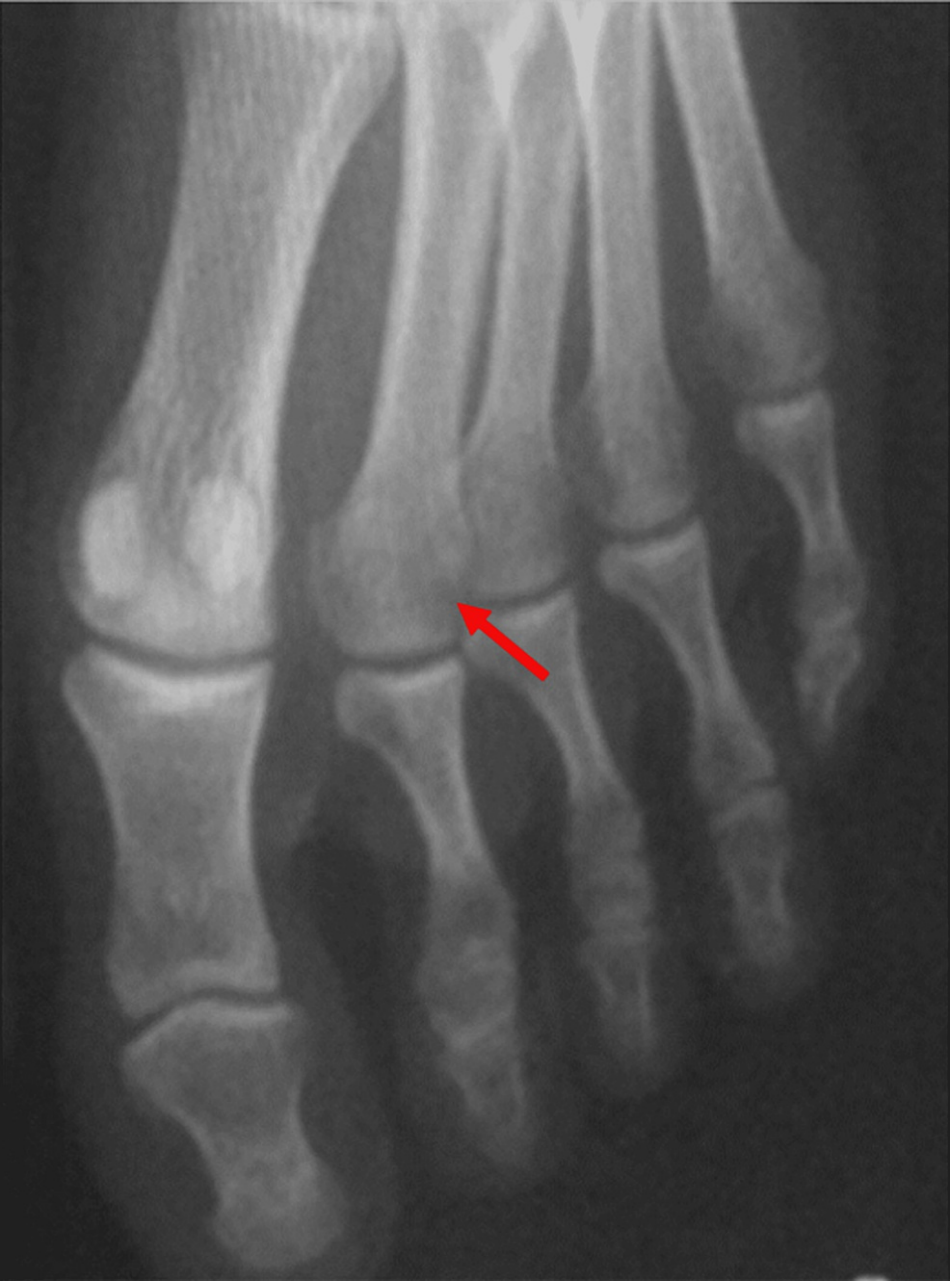
Anteroposterior radiograph three years after the injury showing fracture union without evidence of arthritic changes or aseptic necrosis (red arrow).

Postoperative radiographs confirmed the anatomic reduction of the articular fragment. Total weight-bearing using Barouk shoes was allowed for six weeks. The K-wires were removed after three months, and the patient was able to walk normally without pain and had a complete range of motion of the second MTPJ. He resumed his sport activity four months after the surgical treatment. At the three-year follow-up, radiographs were satisfactory (Figure 3) without necrosis or arthritic changes of the second MTPJ.

Case 2: MHOCF associated with a fifth metatarsal neck fracture

**Figure 4:**
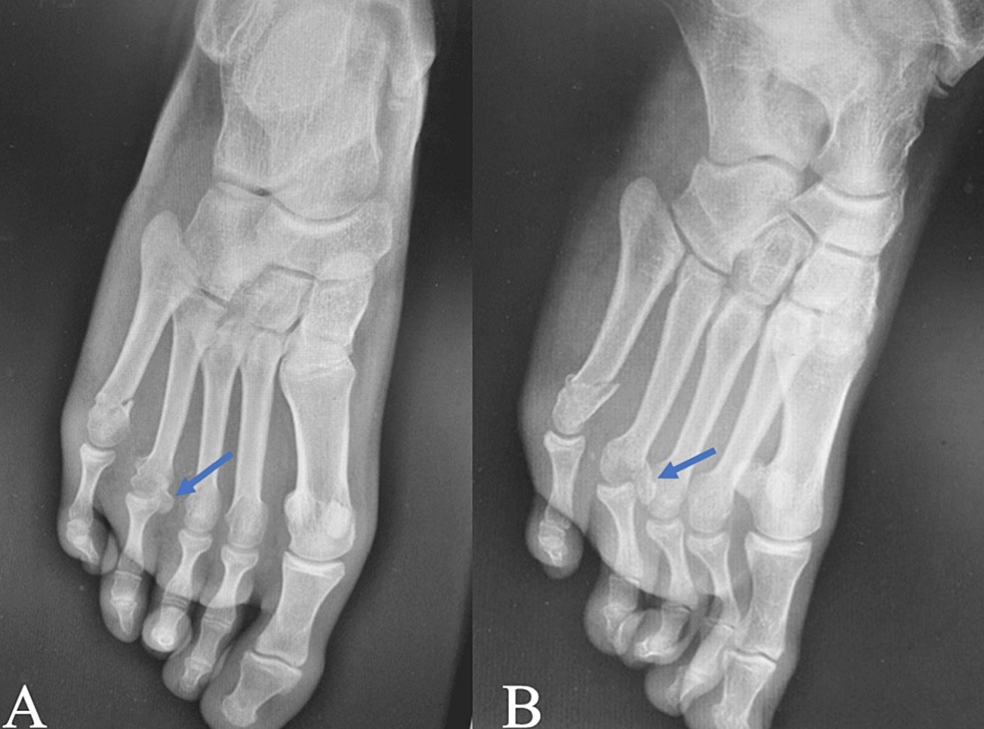
Radiographs of the right foot showing the intraarticular slipped osteochondral fracture of the fourth metatarsal head (blue arrows) associated with a fifth metatarsal bone fracture on both AP and oblique views AP: anteroposterior

A 19-year-old male presented to the emergency department after receiving a right foot trauma secondary to a motor vehicle accident (MVA). He complained of right foot pain and inability to bear weight. Physical examination showed swelling and ecchymosis over the right fourth and fifth MTPJ associated with a limited range of motion. Radiographs of the right foot showed a dorsally displaced fourth MHOCF associated with a minimally displaced fifth metatarsal neck fracture (Figure 4).

Under spinal anesthesia and using the dorsal approach over the fourth web space, the joint was exposed between the extensor tendons. Open reduction internal fixation of the fourth metatarsal osteochondral fragment was performed using a Herbert screw. Nonsurgical treatment was decided for the fifth metatarsal bone fracture.

**Figure 5:**
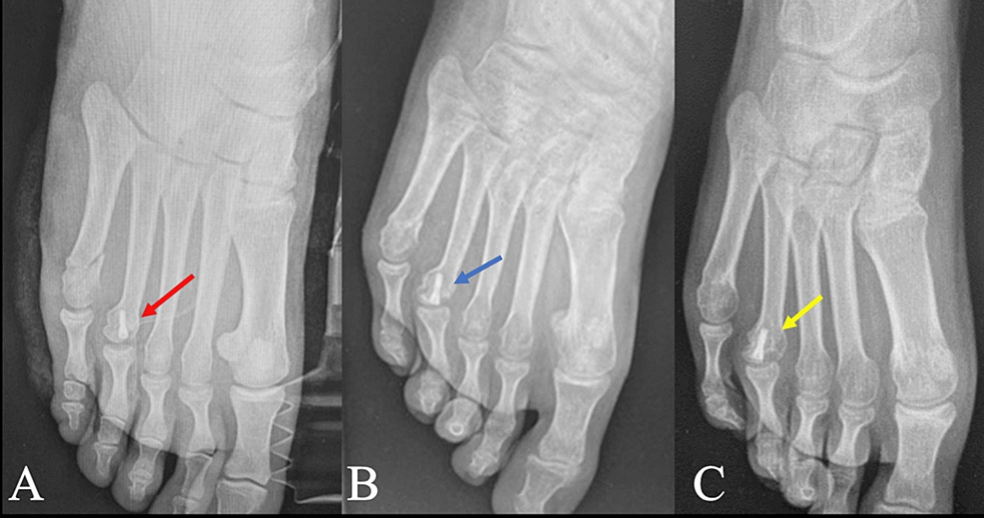
(A) Radiographs of the foot immediately post fixation of the fourth metatarsal head with a Barouk screw (red arrow), (B) eight weeks post-operatively showing a well-consolidated fracture (blue arrow), and three years post-operatively (C) showing a well-consolidated metatarsal head with resorption of the medial part of the head (yellow arrow).

Postoperative X-rays showed satisfactory results (Figure 5A). Total weight-bearing using Barouk shoes was allowed for six weeks. Serial follow-up radiographs showed good screw position with complete bone healing after eight weeks (Figure 5B). At the four-month follow-up, he was allowed to resume sports and daily life activities with no limitations. He had a painless complete range of motion of the fourth and fifth MTPJ. At the three-year follow-up, left foot radiographs (Figure 5C) showed bone healing, with an asymptomatic subchondral lytic bone lesion of the metatarsal head. 

## Discussion

Traumatic MHOCFs are rare injuries. Most of the reported cases in the literature are due to fatigue, stress fractures or Frieberg’s infarction [7]. It is mainly seen in young male patients [6,8], like our two 19-year-old male patients. Several mechanisms have been described to explain this injury. For some authors it results from direct foot trauma [5,8]; whereas, for others, it results from longitudinal stress transmitted to the toe thereby creating a shear force to the metatarsal head responsible for the dorsally displaced retroverted osteochondral fragment [2,7,8].

The first reported case of an isolated traumatic MHOCF was described by Heckman in 1984 [5]. It was a missed untreated fracture with satisfactory functional results despite the persistent dislocation of the MTPJ. Afterwards, two additional cases were reported in 1989 and 1995, by Dutkowsky et al. and Tanaka et al. respectively [2, 8]. These reports increased awareness about this type of fracture. And since 2007, 10 cases were reported successively [1,2,5,6,7,9]. Most of these cases were young male patients (eight out of 13 cases). The second and fourth metatarsal heads were the most commonly affected (five cases each). Diagnostic X-rays of the slipped MHOCF can be sometimes uncertain. In such cases, CT scans are useful to confirm the diagnosis [9].

Management of osteochondral fracture of the metatarsal head is still unclear due to the small number of reported cases in the literature [10]. The goal of treatment is to restore the congruity of the articular surface. Conservative treatment with closed reduction under local anesthesia and immobilization was first described by Dutkowsky et al. who reported acceptable functional results with full passive ROM and 10° flexion loss in active ROM of the third metatarsophalangeal joint [8]. However, failure to reduce the displaced retroverted articular surface can lead to an unsatisfactory clinical and radiological outcome which will need secondary surgical corrections to obtain good results [6].

Open reduction of the articular metatarsal head using the dorsal longitudinal approach was first described by Tanaka et al [2]. Gentle manual traction of the proximal phalanx is recommended to reproduce the articular space for osteochondral reduction and to prevent any damage to the chondral tissues and the blood supply of the poorly vascularized metatarsal head thus avoiding osteochondral infarction [11]. The two-K-wire technique described by Atik et al. for metatarsophalangeal distraction to allow enough articular space for reduction can be helpful to avoid articular surface damage [7]. Internal fixation can be achieved using K-wires, screws, or bioabsorbable pins [1,2,6,7,9,10,12]. Table 3 represents all cases of traumatic MHOCF reported in the literature in addition to the treatment strategy adopted by each author and their respective outcomes [1,2,5-10,12,13].

**Table 1:** Osteochondral metatarsal head fracture reported in the literature. ROM: range of motion; MTP: metatarsophalangeal

Author, year	Age (years), sex	Mechanism of injury	Localization	Treatment	Follow up
Heckman et al. (1984) [5]	Unknown	Not known	Not mentioned	Missed injury	No follow up
Dutkowsky et al. (1989) [8]	18, male	Fall from a tree	3rd metatarsal head	Closed reduction by manipulation and traction Buddy-taped and short leg cast for 6 weeks	8 months, asymptomatic, 10° flexion loss in active ROM, no necrosis, no arthritic changes
Tanaka et al. (1995) [2]	27, male	Jumped during volleyball playing	2nd metatarsal head	Open reduction and internal fixation with a single Herbert screw	25 months, asymptomatic, good ROM, no necrosis, no arthritic changes
Mereddy et al. (2007) [1]	40, male	Kicked on the top of the foot while playing football	4th metatarsal head	Open reduction and internal fixation with a single twist-off screw	12 months, asymptomatic, good ROM, no necrosis, no arthritic changes
Liddle and Rosenfeld (2008) [6]	18, male	Being caught in the handle of a bag, causing forced plantarflexion	2nd metatarsal head	Initially conservative treatment with buddy taping and an Air Cast boot for 5 months then open reduction and internal fixation with two 2.0 mm screws	6 months, minimal pain with weight bearing, good ROM, no necrosis, no arthritic changes
Atik et al. (2013) [7]	19, male	Jumping from a bank on the hard projection of a slipper	4th metatarsal head	Open reduction and internal fixation with 2 cross K-wires	12 months, asymptomatic, full ROM, no necrosis; no arthritic changes
Lui (2015) [9]	32, female	Ran up the escalator	4th metatarsal head	Conservative treatment	25 months, asymptomatic, limitation of plantarflexion
	46, female	Right foot hitting on steps	2nd metatarsal head	Open reduction internal fixation using 2 screws	Plantar protrusion of the screw tip. resulting in painful stiffness of the MTP joint
	14, female	Toe contusion during a football game	4th metatarsal head		Residual stiffness of the MTP joint
	16, male	Direct contusion of his right second toe	4th metatarsal head		No necrosis, no arthritic changes
Temiz et al. (2015) [13]	19, female	Right foot injury after stepping down from higher ground	2nd metatarsal head	open reduction and internal fixation with a headless compression screw	12 months, asymptomatic, full ROM, no necrosis, no arthritic changes
Kurashige and Suzuki (2016) [12]	14, male	Dismounting from his bicycle	3rd metatarsal head	Open reduction, internal fixation with two 1.5-mm bioabsorbable thread pins	12 months, asymptomatic, full ROM, no necrosis, no arthritic changes
Oliveira et al. (2021) [10]	19, male	Left foot trauma when landing from a jump, with the toes in hyperextension.	2nd metatarsal head	open reduction internal fixation using Herbert screw	12 months, asymptomatic, full ROM, no necrosis, no arthritic changes
Current paper	19, male	Left foot trauma during a handball match.	2nd metatarsal head	open reduction internal fixation using k-wire (first case).	3 years, asymptomatic, full ROM, no arthritic changes.
	19, male	Right foot trauma secondary to a motor vehicle accident	4th metatarsal head	open reduction internal fixation using Herbert screw	Lytic subchondral lesion (second case).

The major prognostic factor is the restoration of the MTPJ congruity. So open reduction internal fixation is recommended after failure of closed manipulation to preserve the viability of the distal fragment to prevent the avascular necrosis of the metatarsal head [6]. The main reported long-term complications of surgical treatment are residual pain and mild loss of plantar flexion of the toe.

## Conclusions

Metatarsal head osteochondral fractures are rare fractures that can be easily missed, especially in the emergency department. When suspected and not clearly visible on an X-ray, a CT scan might be essential to confirm the diagnosis and guide surgical treatment. The treatment goal is to achieve the anatomical reduction of the articular fragment to prevent early degenerative changes in the metatarsophalangeal joint that can lead to chronic pain and can interfere with the gait of patients. When this fracture is displaced, open reduction and internal fixation with anatomical reduction is the target to be achieved. K-wires and Barouk screws are both valid options for fixation depending on the size of the osteochondral fragment.
